# Preclinical Evaluation of Sodium Selenite in Mice: Toxicological and Tumor Regression Studies after Striatum Implantation of Human Glioblastoma Stem Cells

**DOI:** 10.3390/ijms221910646

**Published:** 2021-09-30

**Authors:** Louis Larrouquère, Sylvie Berthier, Benoit Chovelon, Catherine Garrel, Véronique Vacchina, Hugues Paucot, Jean Boutonnat, Patrice Faure, Florence Hazane-Puch

**Affiliations:** 1 Medical Oncology Department, Centre Léon Bérard, 69000 Lyon, France; louis.larrouquere@lyon.unicancer.fr; 2 Cytometry Platform, Institute of Biology and Pathology, Grenoble Alpes Hospital, 38000 Grenoble, France; SBerthier@chu-grenoble.fr (S.B.); JBoutonnat@chu-grenoble.fr (J.B.); 3 Unit of Anatomopathology, Institute of Biology and Pathology, Grenoble Alpes Hospital, 380000 Grenoble, France; 4 Unit Nutritional and Hormonal Biochemistry, Institute of Biology and Pathology, Grenoble Alpes Hospital, 38000 Grenoble, France; BChovelon@chu-grenoble.fr (B.C.); CGarrel@chu-grenoble.fr (C.G.); PFaure@chu-grenoble.fr (P.F.); 5 Department of Molecular Pharmacochemistry, University Grenoble Alpes, CNRS, UMR 5063, 38000 Grenoble, France; 6 Ultra Traces Analyses Aquitaine, UT2A, 64000 Pau, France; veronique.vacchina@univ-pau.fr; 7 University of Pau & des Pays de l’Adour, FORCO, Bâtiment d’Alembert-Rue Jules Ferry, BP 27540-64075 Pau CEDEX, France; hugues.paucot@univ-pau.fr

**Keywords:** glioblastoma, cancer stem cells, sodium selenite metabolism and absorption, xenograft, tumor regression, cell death

## Abstract

Glioblastoma (GBM) is the most aggressive malignant glioma, with a very poor prognosis; as such, efforts to explore new treatments and GBM’s etiology are a priority. We previously described human GBM cells (R2J-GS) as exhibiting the properties of cancer stem cells (growing in serum-free medium and proliferating into nude mice when orthotopically grafted). Sodium selenite (SS)—an in vitro attractive agent for cancer therapy against GBM—was evaluated in R2J-GS cells. To go further, we launched a preclinical study: SS was given orally, in an escalation-dose study (2.25 to 10.125 mg/kg/day, 5 days on, 2 days off, and 5 days on), to evaluate (1) the absorption of selenium in plasma and organs (brain, kidney, liver, and lung) and (2) the SS toxicity. A 6.75 mg/kg SS dose was chosen to perform a tumor regression assay, followed by MRI, in R2J-GS cells orthotopically implanted in nude mice, as this dose was nontoxic and increased brain selenium concentration. A group receiving TMZ (5 mg/kg) was led in parallel. Although not reaching statistical significance, the group of mice treated with SS showed a slower tumor growth vs. the control group (*p* = 0.08). No difference was observed between the TMZ and control groups. We provide new insights of the mechanisms of SS and its possible use in chemotherapy.

## 1. Introduction

Glioblastoma (GBM) is the most aggressive malignant glioma. Outcomes remain very poor, with a median overall survival of 15 months [1]. The current standard of treatment has been unchanged since 2005, with the use of radiotherapy plus concomitant and adjuvant temozolomide (TMZ) [2]. 

The molecular pathogenesis of GBM shows recurrent driver somatic mutations in PTEN and p53, epidermal growth factor receptor (EGFR) gene amplification, telomerase reverse transcriptase (TERT) promoter mutation, chromosome 7 gain combined with chromosome 10 loss, and homozygous deletions on CDKN2A/B gene loci, all leading to a dysregulation of core pathways such as the receptor tyrosine kinase (RTK) phosphatidylinositol 3′-kinase (PI3K)/AKT axis, p53, and retinoblastoma (RB) pathways [3,4,5]. Recently, Lee et al. demonstrated that astrocyte-like neural stem cells (NSCs) from the sub-ventricular zone (SVZ) could be the cells of origin of GBM; indeed, these NSCs hold some of the driver mutations and migrate to distant brain regions, leading to the development of GBM [6]. This result would sustain the hierarchical model for carcinogenesis, which states that cancer stem cells (CSCs) are the source of tumor formation, metastasis, and relapse. If the debate about the origin of GBM cells has been alleviated since the findings of Lee et al., for other groups, this debate should be refocused on the tumor-spreading cell population [7]. The R2J patient-derived glioblastoma stem cell model that we have developed exhibits CSC properties [8]. In vitro, CSCs from brain tumors were isolated and identified on the basis of their renewal, differentiation, tumorigenesis capability, and cell surface antigen expression [9]. In vivo, CSCs cultivated in gliospheres (R2J-GS) in serum-free media and representing ~1% of the parental cell line can proliferate into deficient animals, and are at the origin of the tumor development when orthotopically grafted [10]. CSCs are a fundamental concept in cancer biology, and the development of therapeutic strategies targeting CSCs has become a hot topic [11]. Many CSC markers—such as Sox2, Olig2, and nestin—have been identified, and are a valuable aid in identifying CSCs, even if they do not constitute a consensus signature. Some of them are therefore chosen to constitute a panel for identifying CSCs, sometimes to the detriment of others. Indeed, according to the review of Brescia [12], CSCs are heterogeneous, and can be characterized by a variety of markers. CD34 is one such marker, and is used not only for glioblastomas, but also for other subtypes of cancer (liver, pancreas, leukemia...) to define CSCs, as well as being used to characterize endothelial cells [13]. In this regard, CSCs are able to differentiate into endothelial cells, which is also called vasculogenic mimicry [14]. It should be noted that nestin appears to be a robust marker according to the literature [15]. 

Selenium is required for the production of selenoproteins (SPs), essential for antioxidant and detoxifying activities, such as sodium selenite (SS), a toxic intermediate of selenocysteine biosynthesis. Recently, it has been shown that selenophosphate synthetase 2 (SEPHS2) is crucial for the survival of cancer cells [16], because it detoxifies selenide. These results underlie the potential therapeutic approach of using toxic Se metabolites, such as SS, to target cancer cells.

SS has been widely studied in vitro and in vivo, and several clinical trials have studied its effects in carcinoma (sodium selenite as a cytotoxic agent in advanced carcinoma (SECAR)) and in prostate cancers in association with abiraterone and prednisone (NCT04296578). The SECAR study determined the maximum tolerated dose (MTD) at 10.2 mg/m² [17], and a phase II study is ongoing.

Previous works, including ours, have tried to elucidate the molecular mechanism underlying the anticancer effects of SS. We have shown that SS induces an oxidative stress indirectly measurable via the depletion of thiol groups in GBM cell lines [8]. The generation of reactive oxygen species (ROS) induced by SS has been established in diverse cancer cell lines, causing multiple dysregulations, such as an upregulation of β-catenin pathway, itself targeting genes such as cyclin D1 in colorectal cancer cells [18]. In NB4 cells, SS leads to cell cycle arrest and apoptosis via the generation of hydrogen peroxide, leading to the regulation of the JNK/ATF2 axis and the inhibition of cyclin D3 [19]. In vivo, the injection of NB4 cells into nude mice treated with SS every 2 days at 3 mg/kg/day for 3 weeks led to more dead cells in SS-treated tumor tissues vs. untreated mice, confirming the authors’ published in vitro results [19]. 

Temozolomide (TMZ) is an oral alkylating agent used to treat glioma, and particularly GBM. However, its efficacy depends on MGMT gene promoter methylation, and at least 50% of GBMs do not have the MGMT gene promoter (hMGMT) methylated [20]. In this population, outcomes are worse than those of other GBMs due to the decreased response to TMZ. 

It is then essential to find alternative therapies to be combined (or not) with the existing ones, so as to trigger different mechanisms of action and sensitize cells to TMZ. The use of TMZ combined with SS could be interesting, as we previously showed that both drugs activated different mechanisms of action in vitro. Indeed, unlike SS, TMZ did not induce oxidative stress or a disruption of the redox status. Autophagy was induced by SS, but not by TMZ, in our experimental conditions. Consequently, the combination of both drugs may synergize to induce cell death in GBM cell lines [8]. 

Collectively, all of these findings—i.e., validation of the R2J-GS orthotopic xenografted model of GBM and SS as an attractive agent for cancer therapy—led us to test the effects of SS on tumor growth in R2J-GS cells orthotopically implanted in immunodeficient mice, and to further characterize the molecular mechanisms involved in SS toxicity. 

## 2. Results

### 2.1. In Vitro Study

#### 2.1.1. R2J-GS Characteristics and Plasticity in Culture

**Figure 1:**
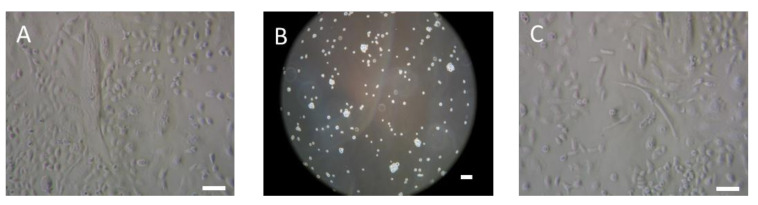

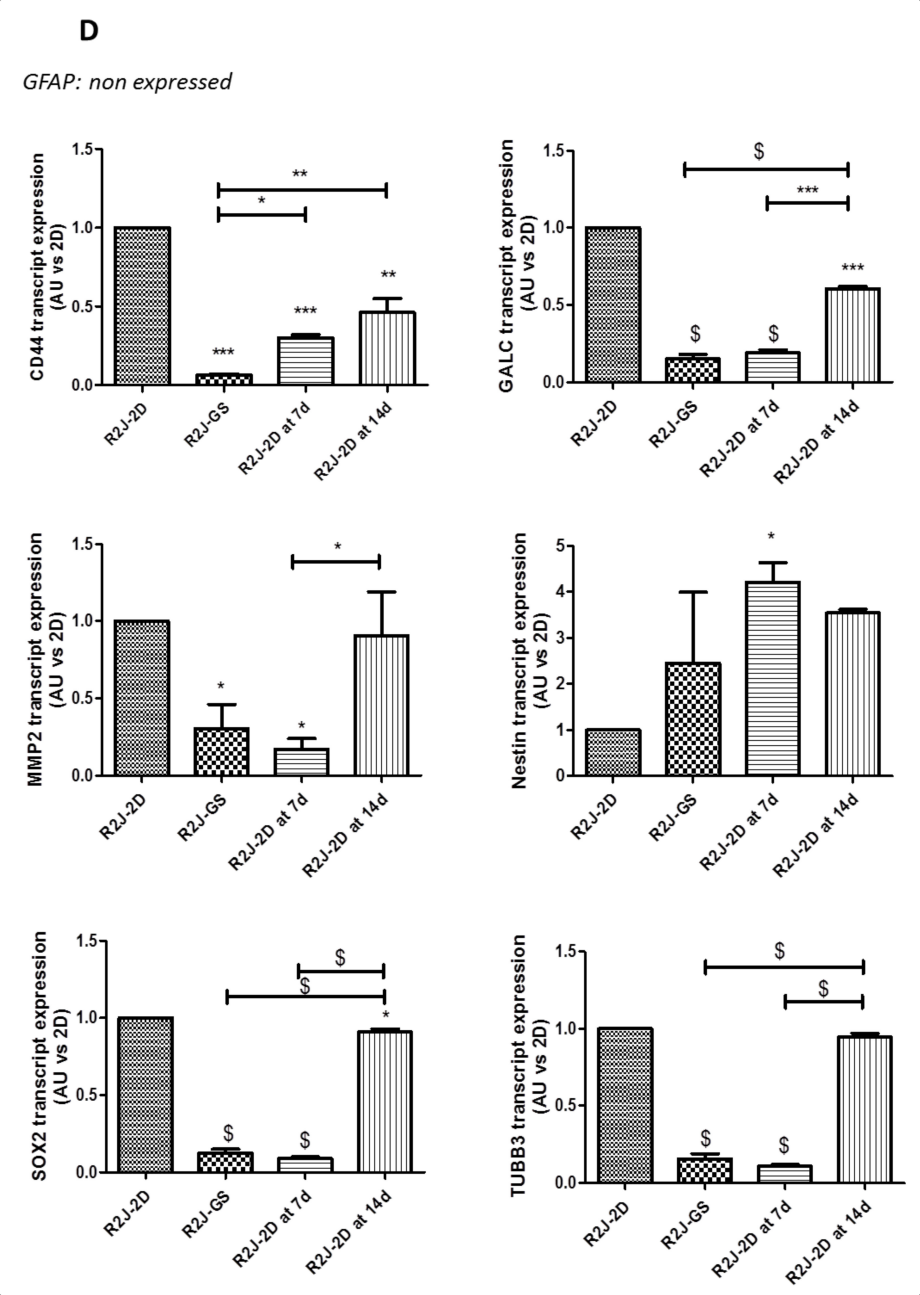
R2J cells were plated in monolayer (**A**) in serum-enriched medium (10%) at 6000 cells/cm², harvested, and seeded at 6000 cells/cm² in media without serum in a non–adherent 10 cm Sarstedt dish. Seven days later, R2J-GS cells (**B**) were harvested and plated again in monolayer conditions as described above. R2J cells (**C**) were harvested with trypsin 7 or 14 days after the plating to evaluate (**D**) their molecular plasticity via RT-q-PCR. Pictures were taken at 300× (**A**,**C**) and 100× (**B**), with an optical microscope. Scale bar = 100 µm. * *p* < 0.05; ** *p* < 0.01; *** *p* < 0.005, and $ *p* < 0.0001.

To study the molecular plasticity of R2J-GS cells via RT-q-PCR, the cells were alternatively cultured in monolayer (Figure 1A) and then in gliospheres (Figure 1B), harvested, and plated again in monolayer for 7 and 14 days (Figure 1C). 

In non-adherent and serum-free conditions, R2J-GS cells were a self-renewing and highly proliferative population. When plated again in serum-enriched medium (Figure 1C), R2J-2D cells again acquired their phenotypic heterogeneity, i.e., giant, fibroblastic-like, and astrocytic-like cells (Figure 1A,C).

The expression of CD44, GALC, MMP2, SOX2, and TUBB3 was depleted under the GS state, and progressively restored 14 days after culture in monolayer with serum (Figure 1D), except for nestin, which was slightly augmented in GS and significantly increased in monolayer at 7 days, and tended to decrease at 14 days in 2D. 

Then, the phenotype acquired in monolayer and serum conditions was progressively reversed in GS, and needed almost 14 days to be restored in monolayer and serum conditions.

#### 2.1.2. Se Was Uptaken and Metabolized in Se(0) by R2J-GS 

Se uptake by R2J-GS cells was evaluated by ICP–MS measurement 24 h after treatment. 

**Figure 2:**
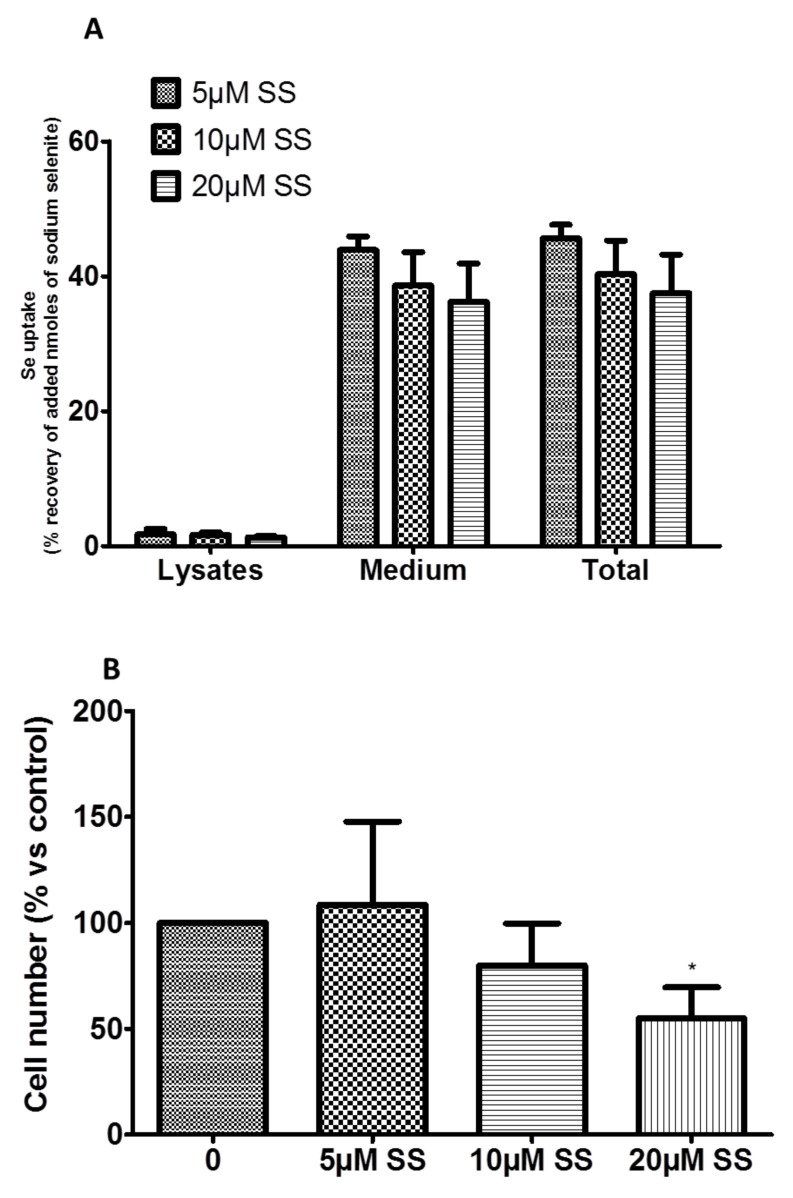

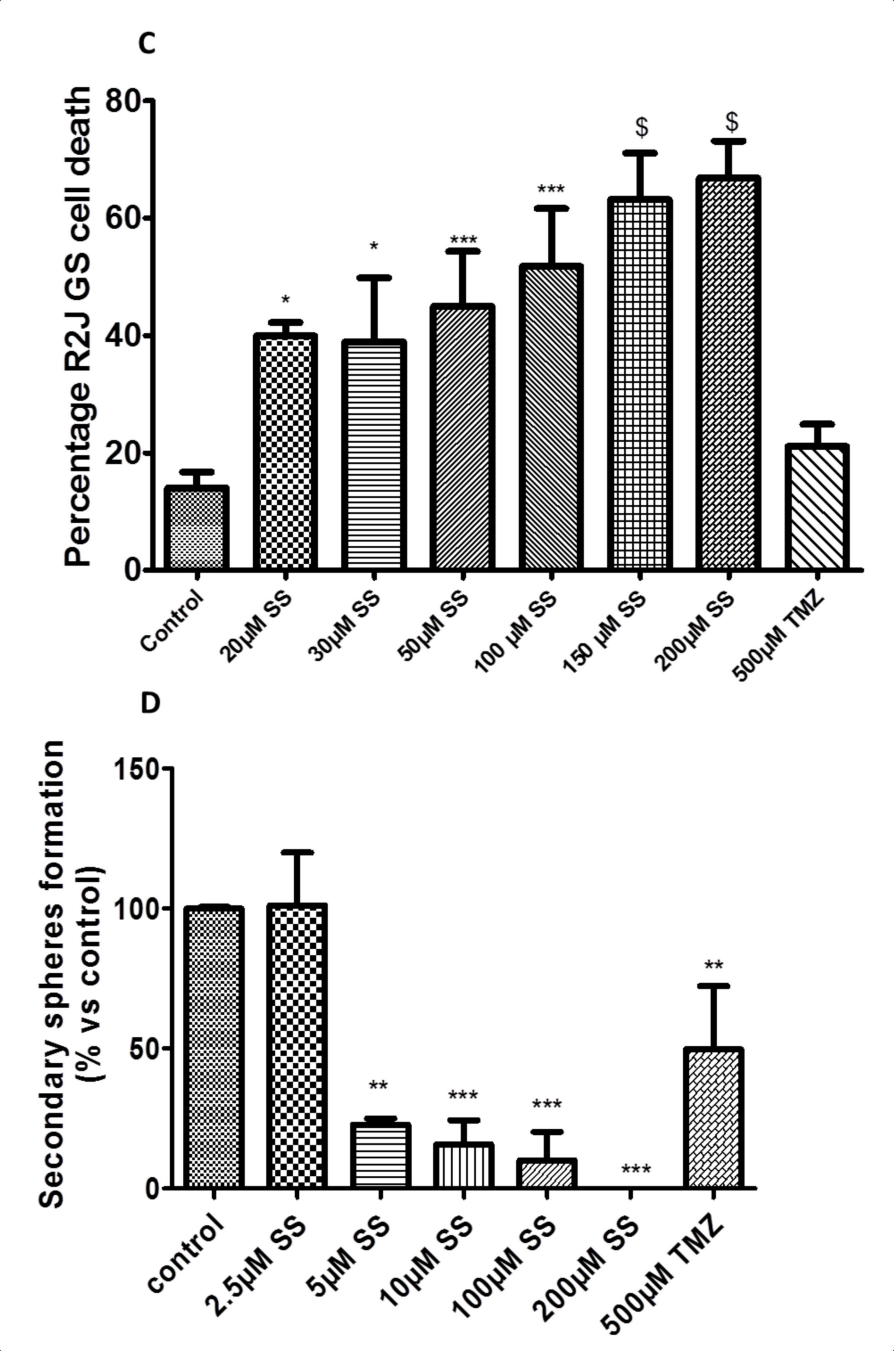

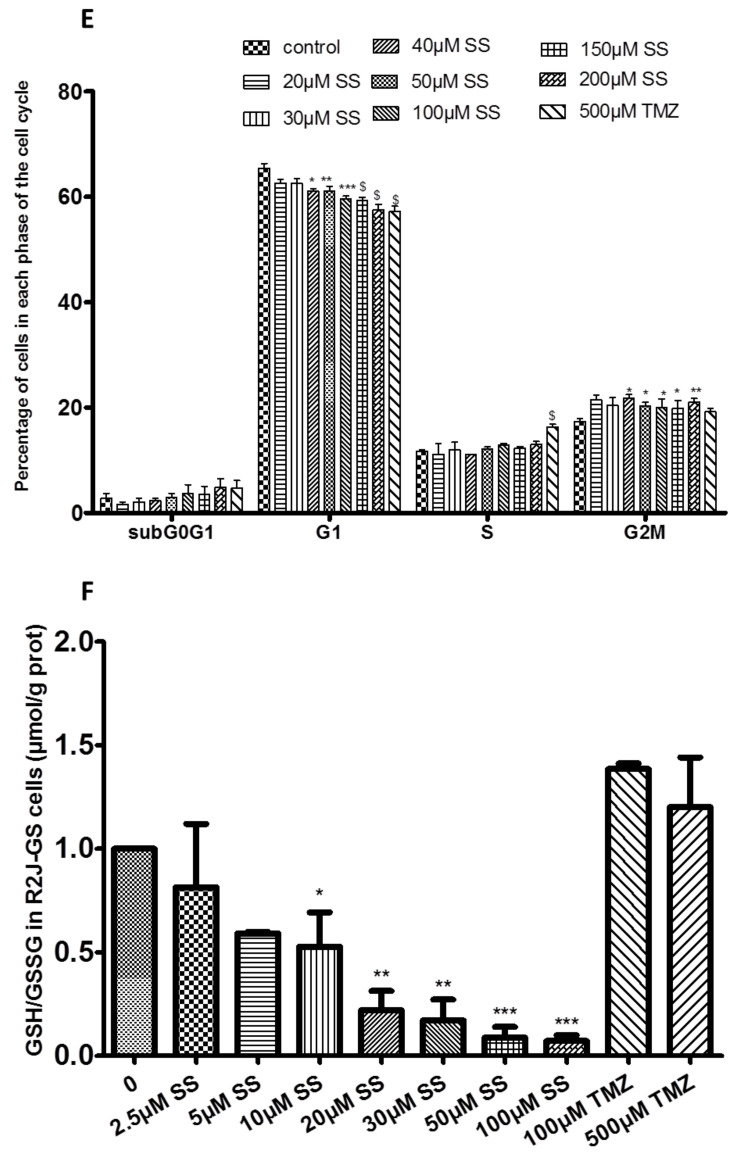
Absorption of Se; biochemical analysis and toxicity of SS and TMZ in R2J-GS cells. (**A**) Selenite uptake by R2J-GS cells. R2J-GS cells were treated for 24 h with SS at 5, 20, or 40 µM. Se was determined via ICP–MS. The quantity recovered was calculated on the basis of the quantity supplied by the treatment. (**B**) Invasion of R2J-GS cells was evaluated via transwell assay. Cells were seeded on a collagen I matrix in the upper well and treated for 24 h with different concentrations of SS. The following day, cells that migrated to the bottom well were counted. (**C**) Both necrosis and apoptosis in R2J-GS cells after 24 h of SS treatment were determined via flow cytometry after Annexin-V and IP staining. (**D**) Secondary sphere formation was assayed via soft agar assay. After SS or TMZ treatments, R2J-GS cells were allowed to grow for 14 days in soft agar. Colonies were then counted after crystal violet staining, under an optical microscope. (**E**) The cell cycle in R2J-GS cells was evaluated via flow cytometry after 24 h of treatment with different doses of SS. DNA damage was deducted by the percentage of cells in the subG0G1 phase. (**F**) Oxidative stress was evaluated by the GSH/GSSG ratio in R2J-GS cells after SS and TMZ treatments for 24 h. * *p* < 0.05; ** *p* < 0.01; *** *p* < 0.005, and $ *p* < 0.0001.

Whatever the SS concentration added, the differences were not significant with regard to the cell compartment considered (Figure 2A). The intracellular Se uptake remained low (mean ± SD = 1.6% ± 0.2), whereas the majority was found in the medium (mean ± SD = 39.6% ± 3.9). The mean ± SD total recovery was then 41.1% ± 4.1, meaning that more than half of the added Se was lost—probably due to volatile Se metabolites, as previously noted [21]. 

As the cell pellets were red, we hypothesized that R2J-GS cells were able to metabolize SS in amorphous selenium (Se0). This metabolism is well described in bacteria, but relatively unknown in mammalian cells. Elemental selenium was detected following treatment with sulfite, which resulted in the formation of selenosulfate anions, confirming our hypothesis and allowing us to quantity the Se(0) formed in cells. Indeed, at 20 µM and 40 µM SS treatment, R2J-GS cells metabolized 2.73% ± 0.40 and 1.44% ± 0.04 of the SS in Se(0), respectively.

#### 2.1.3. SS Decreased Cell Invasion and Induced Both Apoptosis and Necrosis in R2J-GS Cells

The number of R2J-GS cells counted in the lower well decreased (significantly at 20 µM SS over 48 h) with the concentration of SS added to the upper well (Figure 2B).

SS induced significant cell death at 20 µM treatment for 24 h (Figure 2C). It is worthy of note that SS’s IC50 was 107.9 µM ± 1.2 vs. 3.2 µM ± 0.2 in R2J cells cultivated in monolayer and in serum conditions [8], meaning that R2J-GS cells are more resistant to SS than R2J cells. R2J-GS cells are also resistant to TMZ, as at 500 µM for 24 h, 80% of cells survived. Greater TMZ doses were not tested due to the toxicity of DMSO.

The formation of secondary spheres in soft agar after treatment with 20 µM SS for 24 h was significantly inhibited (Figure 2D).

#### 2.1.4. SS Induced Cell Cycle Arrest in R2J Spheres

Although R2J-GS cells were quite resistant to SS treatment, they were arrested from 40 µM SS in the G2M phase of the cell cycle (Figure 2E). It should be noted that R2J cells cultivated in monolayer and serum conditions were also blocked in G2M, but from only 2.5 µM SS [8].

#### 2.1.5. SS Induced Oxidative Stress in R2J Spheres

The oxidative stress induced by SS was evaluated by the determination of the GSH/GSSG ratio in R2J-GS cells after 24 h of treatment (Figure 2F). At 10 µM SS treatment, we noticed a significant decrease in the ratio, whereas TMZ did not change it. 

### 2.2. In Vivo Study: Evaluation of SS Toxicity

#### 2.2.1. Until 6.75 mg/kg, the Mice Did Not Exhibit Signs of Toxicity 

BALB/c mice orally received SS at the indicated doses (n = 3 mice/dose) according to the experimental design detailed above. The mice were weighed daily. 

We did not see any signs of abnormal behavior or notable toxicity until 6.75 mg/kg, whereas at 10.125 mg/kg a significant weight decrease was noted—probably partly due to digestive toxicity (data not shown); indeed, at the dissection step, we observed that the gastrointestinal tract was hemorrhagic. At 10.125 mg/kg, the mice had yellowish hair and cleaned themselves less, while the other groups of mice maintained silky hair and behavior consistent with the control group.

**Figure 3:**
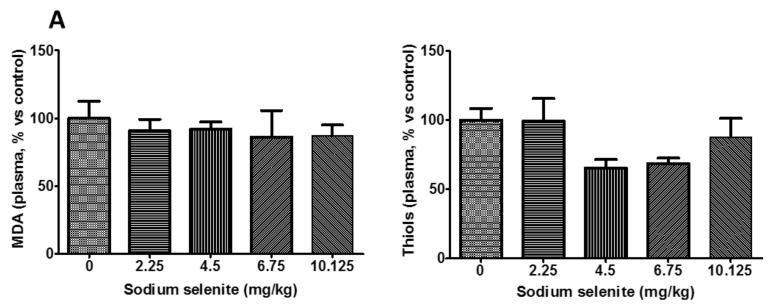

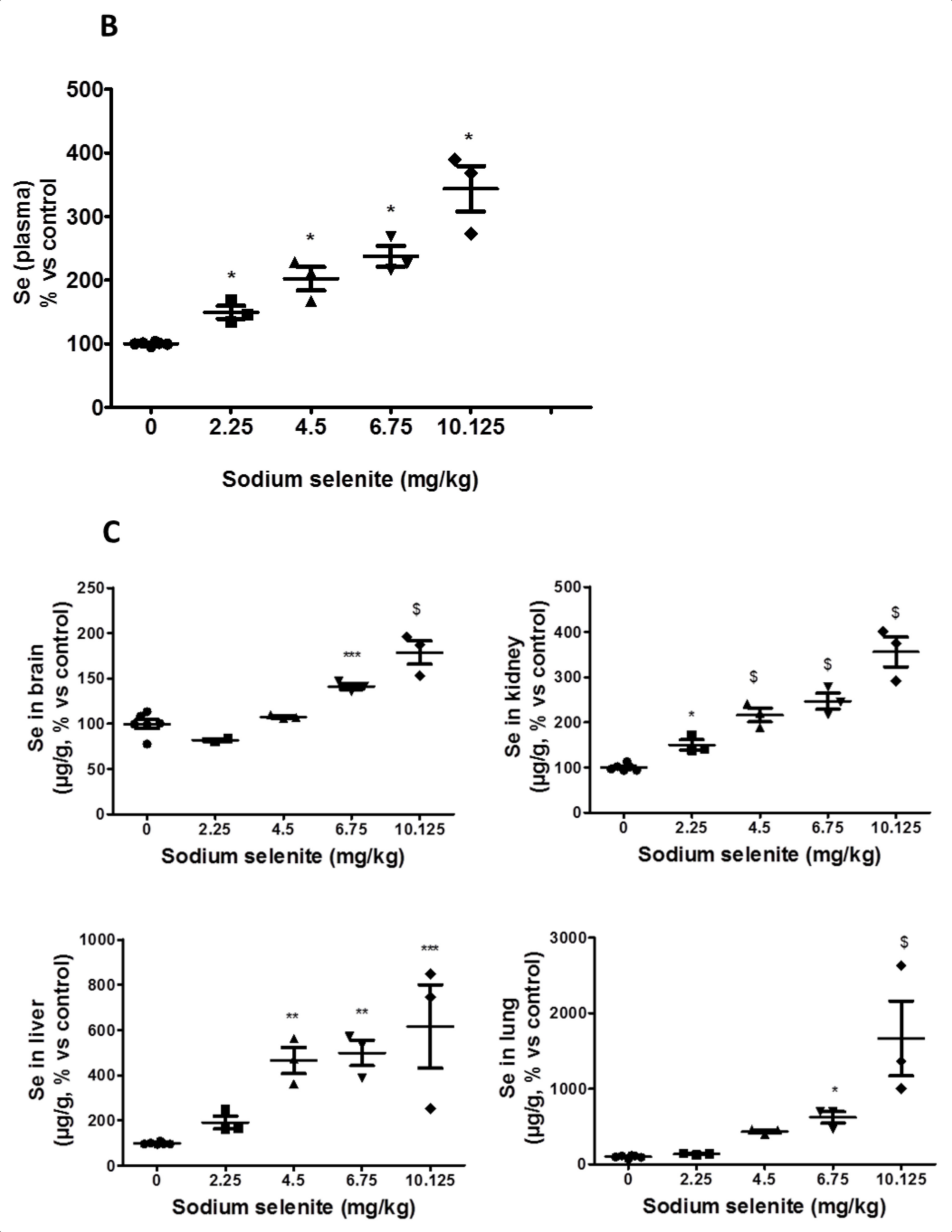

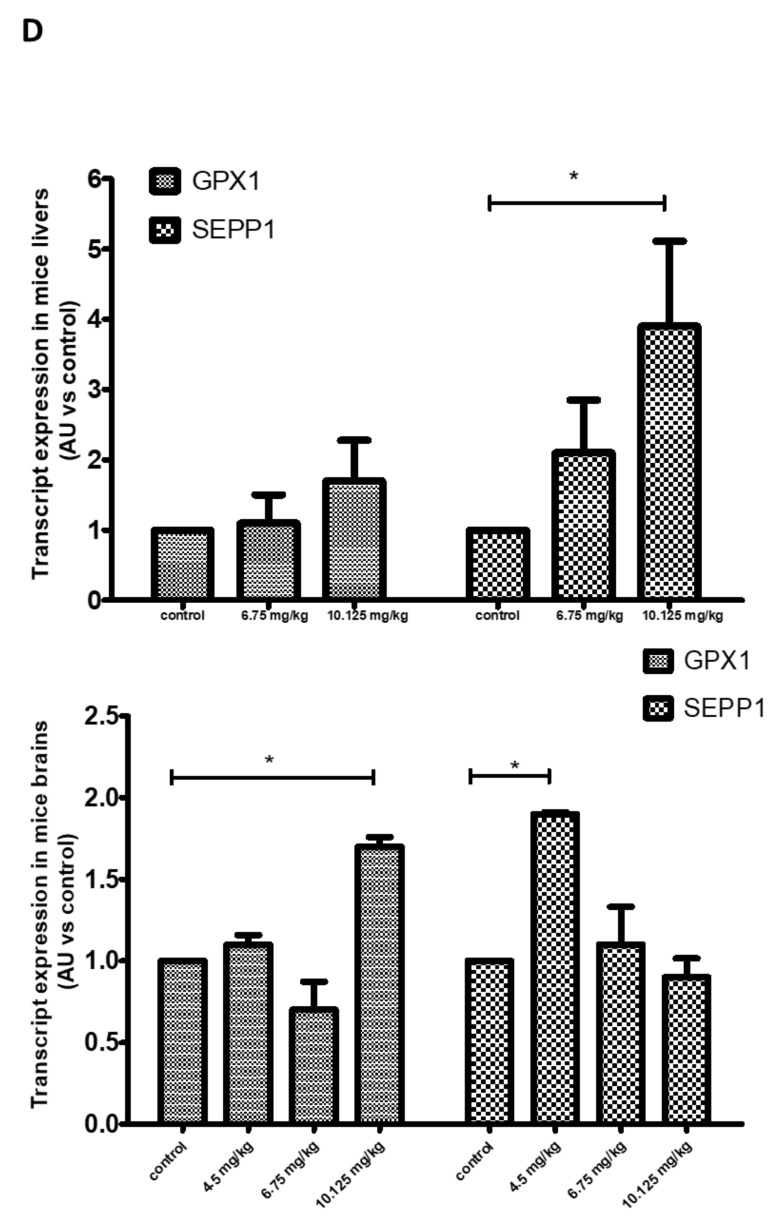
Evaluation of SS toxicity and absorption to evaluate Se status. (**A**) Plasmatic oxidative stress markers of mice receiving SS orally, at different doses (n = 3 mice/concentration group). The dosage of Se was measured in (**B**) plasma and (**C**) in organs via ICP–MS in mice. (**D**) The transcript expression of GPX1 and SEPP1 was evaluated in the brains and livers of mice receiving SS orally. * *p* < 0.05; ** *p* < 0.01; *** *p* < 0.005, and $ *p* < 0.0001.

Interestingly, several of the plasmatic markers of oxidative stress were impacted, e.g., MDA, thiols (Figure 3A). Moreover, SS supplementation did not change glutathione peroxidase-1 activity (GPX1), albumin concentration, or the GSH/GSSG ratio in the liver (data not shown).

#### 2.2.2. Se in Plasma and Organs Were Found at Different Levels

The augmentation of plasmatic Se was linear regarding the concentration of SS (Figure 3B). In the control group, the plasmatic concentration was ~5 µM—i.e., 89 µg/L vs. 125 µg/L in humans—at baseline.

Se was found in all of the organs tested, but the distribution among tissues differed as follows: lung > liver > kidney > brain (Figure 3C). 

Interestingly, Se was significantly found in the brain at 6.75 mg/kg, and these results, combined with the absence of notable toxicity at this dose, led us to choose it to perform a tumor regression study.

#### 2.2.3. Transcript Expression in SS-Supplemented Mice

In brains, GPX1 was significantly increased at 10.125 mg/kg, while SEPP1 was increased at only 4.5 mg/kg.

In livers, GPX1 was unchanged with SS supplementation, and SEPP1 only significantly increased at 10.125 mg/kg.

### 2.3. In Vivo Study: Tumor Regression Assay

#### 2.3.1. Evolution of Neuronal and Stem Cell Markers after SS and TMZ Treatment in Mice Implanted with R2J-GS Cells

**Figure 4:**
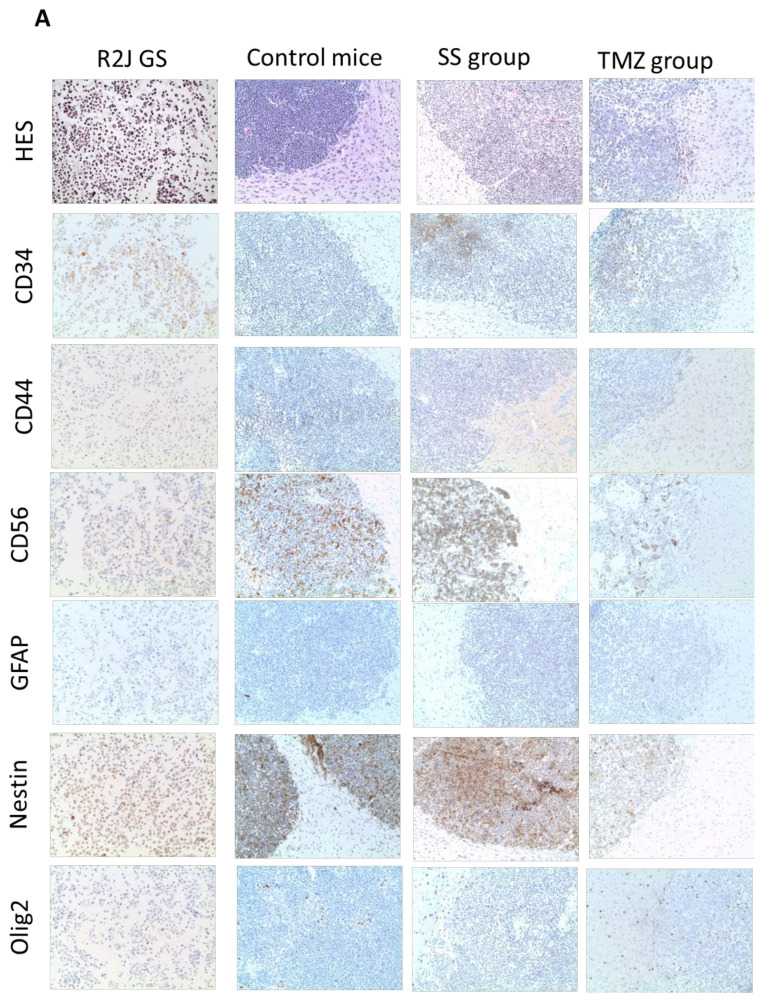

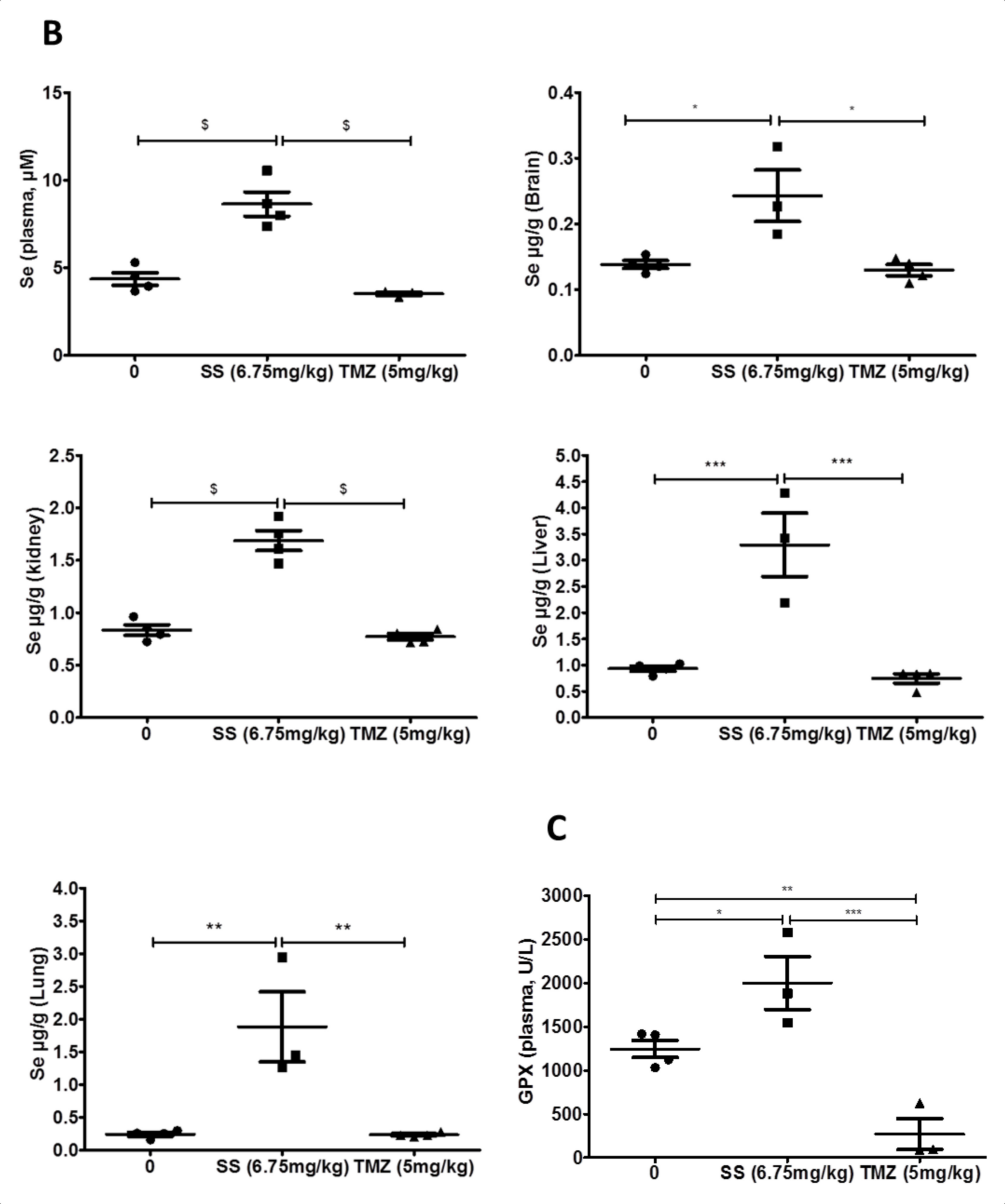

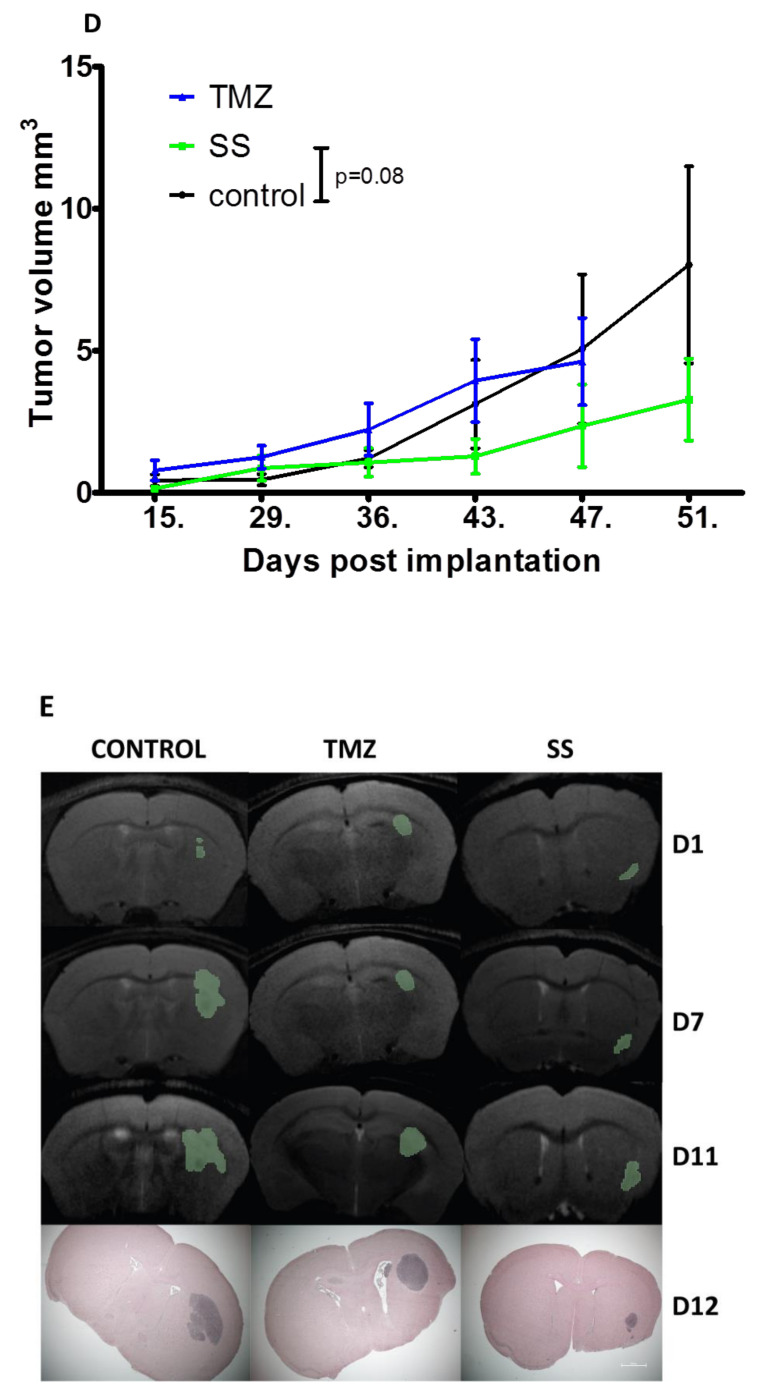
(**A**) Immunocytochemical staining for CD34, CD44, CD56, GFAP, nestin, and Olig2 expression in R2J-GS cells in culture just before their implantation into the striatum, and in the control mice group (placebo-receiving group), SS group, and TMZ group. Scale bar = 100 µM; objective X20. Evaluation of (**B**) Se in plasma and in the brain, kidneys, liver, and lungs, and (**C**) GPX activity in mice receiving SS and TMZ orally compared to the control group. (**D**) Mice were implanted with 1000 R2J-GS cells, were divided into three groups (control, SS receiving 6.75 mg/kg, and TMZ receiving 5 mg/kg), and the progression of the tumors was followed by MRI 15, 30, 37, 41, 47, and 51 days post-implantation. Tumor volume was calculated using 3D Slicer software, and comparisons were performed between the control vs. SS and control vs. TMZ groups. (**E**) Representative coronal MRI image of the tumor volume (green area) during treatment, and HES staining of the coronal section of the whole brain. Scale bar = 500 µm. * *p* < 0.05; ** *p* < 0.01; *** *p* < 0.005, and $ *p* < 0.0001.

After the treatment with SS at 6.75 mg/kg or TMZ at 5 mg/kg, neuronal lineage (CD56, GFAP, Olig2) or stem cell expression markers (CD34, CD44, nestin) were studied via IHC and compared to R2J-GS cells’ profile at the moment they were implanted (Figure 4A). 

All of the markers were expressed in R2J-GS cells at the moment of implantation, except for GFAP and CD44, as already mentioned. In the tumor cells, GFAP and CD44 expression were not recovered under SS or TMZ treatments.

CD34, CD56, nestin, and Olig2 were expressed, but their expression levels did not differ regardless of the experimental conditions, meaning that neither SS nor TMZ treatments affected the expression of these markers. 

#### 2.3.2. Increase in Se in Plasma and Organs of Mice Receiving SS Orally

The dosage of Se in plasma and in organs confirmed that Se circulated in the plasma and was retained in the brain, lungs, liver, and kidneys, with the same distribution affinity as in the toxicity study, at 6.75 mg/kg (liver > lung > kidney > brain), whereas it did not vary in the TMZ group (Figure 4B).

The treatment with SS induced a significant increase in GPX activity in plasma, which was not observed in the toxicological study at 6.75 mg/kg, whereas the baseline values were comparable (mean ± SD, U/L, 1244.0 ± 113.9 vs. 1896.0 ± 594.4, respectively). It is worth questioning whether the implantation plus the SS treatment can influence antioxidant responses. We observed that GPX was significantly decreased in the plasma of mice treated with TMZ (Figure 4C). 

#### 2.3.3. Evolution of the Xenograft Tumor as a Function of the Treatment (SS vs. TMZ): Clinical Responses

The tumor volume was calculated after each MRI and reported as a function of time (Figure 4D). As the sizes of the tumors were heterogeneous, we had to create three treatment groups with matched tumor sizes. Consequently, the treatment of the TMZ group began one week before that of SS group. Then, because of this lag, it was not statistically possible to compare the SS and TMZ groups over the course of the treatment.

It appears that—although not reaching the level of statistical significance, due to the small size of each group—the group of mice treated with SS showed a slower tumor growth than the control group (*p* = 0.08). No difference was observed between the TMZ and control groups. Our tumor xenograft model shows the formation of a bulk tumor with some degree of invasiveness. Indeed, tumor cells spread across the tumor front, as shown in Figure 4E, i.e., tumor evolution under treatment (representative coronal MRI image of the tumor volume (green area) during treatment) and HES staining of the coronal section of the whole brain.

## 3. Discussion

The failure of several cytotoxic therapies in vitro and in preclinical studies is a serious reminder of the complexity and heterogeneity of GBM cells. Here, we provide new insights into the mechanisms of SS and its possible use in chemotherapy. Our results, combined with SS’s apoptotic effects, inhibition of proliferation and invasion, and encouraging preclinical results, support the antitumor properties of SS.

Indeed, functional characteristics are required, such as self-renewal [22], persistent proliferation, and tumorigenesis [10]. Interactions between all identified stem cell markers are preponderant in establishing a stem cell phenotype, and it is therefore difficult to consider any one of them independently of the others. For example, when PARK7—involved in the maintenance of the GBM stem cell strain—is knocked down, the expression of nestin decreases, along with that of EGFRvIII, SOX2, OCT4, NANOG, NOTCH1, non-phospho-β-catenin, Musashi1, and BMI-1, as well as the ability to form spheres [23]. It is known that Notch-1 promotes nestin expression in glioma cells and inhibits their differentiation, so the involvement of Notch-1 in R2J-GS cell activity would also be of interest to better understand the workings underlying the expression of these markers.

R2J cells cultured in gliospheres in non-adherent medium conditions are a self-renewing and highly proliferative population. They express cancer stem cell markers, such as CD34, MMP2, nestin, and SOX2, as well as the astro-neuronal lineage markers GALC, TUBB3 (CD56), and OLIG2. These molecular markers are part of the characteristics of CSGs [24] and there is some plasticity between the different cell states imposed by cell culture conditions, such as oxygen exposure [25], which allows interconversion between CSG and non-CSG states.

Our results are not consistent with those of Lee et al. showing that the culture in adherent plates had reduced cellular differentiation compared to the culture in GS [26]. The method of cultivating cells, the materials used, and the oxygen exposure can impact the transcript expression [27]. Nestin and SOX2 are implicated in repressing differentiation, in supporting stem-like proliferative phenotypes, and in the GMT process [28,29]. It should be noted that the phenotypic heterogeneity of R2J cells was found again after re-plating in 2D. There was then an association between transcript expression and phenotype, suggesting that R2J cells would be able to differentiate into a specific neuronal lineage. Indeed, after the removal of growth factors (EGF and FGF) and the addition of serum, R2J cells began to gain GSC markers, developing morphologies and transcript expression consistent with cells of glial and neuronal lineages.

For CD44, it is worth noting that protein expression was lost in implanted R2J-GS cells, which was not the case in our previous study, meaning that this cell line is still evolving. The same was true of TUBB3, which was previously not detected in R2J-2D cells, whereas it was expressed in R2J-GS cells. Herein, TUBB3’s transcript expression pattern was subsequently dropped in GS and serum-free conditions, and restored under monolayer and serum conditions. 

R2J cells of the GS phenotype were 33-fold more resistant than those of the 2D phenotype, meaning that they acquired a more resistant state, which is also a hallmark of CSCs [29], and is linked to a mesenchymal-like state [30]. Carro et al. showed an altered DNA methylation pattern in resistant clones—particularly in genes regulating the mesenchymal transformation [31]. Cusulin et al. classified GSCs into two groups, and R2J-GS cells fill the criteria for the progenitor-like subtype, expressing a mesenchymal signature and shorter survival when grafted in animals. We previously observed that our mice implanted with 2.10^5^ R2J-GS cells had to be euthanized at 32 vs. 47 days PI with 1000 cells, which is also why we decided to xenograft 1000 R2J-GS cells, i.e., to ensure a relevant timespan in which to test the treatment. 

Adverse effects of SS are known, including hair loss, fatigue, nausea, and vomiting as reported both in humans [17] and in mice after gavage with SS [32]. The toxicity of SS is then the main concern to deal with, and several groups have tried to limit it using different strategies. One approach consisted of combining SS with lentinan—a polysaccharide carrier; the authors showed that the toxicity of SS was reduced without losing its antitumor effects following IP injection in BALB/c mice, which developed tumors after receiving B16-BL6 cells [33]. Nevertheless, some in vitro [34] and in vivo studies showed that SS exhibited less toxicity against normal cells compared with tumor cells, and more affinity [35].

The metabolism of SS in Se(0) is now known, and was reported in an in vivo study: in rats supplied with SS at 0.5 mg/kg/d for 28 days, Se(0) was found in the liver, kidneys, and feces [36]. It has long been known that SS is reduced via its interaction with glutathione [37]. It is now recognized that the depletion of the natural reductive pathway of SS by glutathione (GSH) leads to the generation of Se(0) via the formation of unstable selenodiglutathione (GSSeSG) or selenopersulfide intermediates [38], which produce superoxide and hydrogen peroxide, participating in oxidative damage and in SS toxicity. It has also been shown that, in mammals, the reduction of SS to Se(0) is realized by the gut microflora [39]. We can then question the role of Se(0) in cell toxicity. An interesting study by Jimenez-Lamana [40] showed that nanoparticles of Se (Se(NP)) are at least partially composed of Se(0), implying a link between Se(0) and Se(NP). Another work, studying the toxic effects of Se(NP) in A-172 GBM cells, showed that a weak concentration of Se(NP) induced apoptosis associated with changes in the redox status and ER stress in these cells [41]. 

The metabolism of selenite to Se(0) and its cellular effects, including toxicity, remains a poorly understood aspect of its mode of action and therefore deserves further investigation.

The import of cysteine by xCT (cysteine/glutamate antiporter) causes an intracellular reduction to cysteine, and the following export increases the extracellular reduction capability in the form of the reduced thiol groups from cysteine, which are involved in Se uptake [42], via SLC7A11 [16]. Then, the subsequent reduction of SS to selenide through this selenocysteine biosynthesis pathway is essential for SS toxicity. R2J-GS cells may be low-xCT cancer cells, as the uptake of Se remained low (<2%) combined with a limited effect on selenoprotein expression. Indeed, GPX1 transcript was increased in the brains of mice supplemented with 10.125 mg/kg vs. no change in the liver, whereas SEPP1 was augmented in the brain at 4.5 mg/kg and in the liver at 10.125 mg/kg. The discordant data of GPX activity in plasma between toxicological and regression studies cannot be attributed to variation in Se content or to an impaired Se uptake, as baselines were comparable between both studies. Nevertheless, this significant increase in the regression study must be taken into consideration, as GPX is implied to play a role in the detoxification system. Thus, such an increase may strongly contribute to tumor resistance against oxidative stress caused by SS, and should be taken into account in personalized therapy [43].

Selenite was found to enter the cells in trace amounts, which were nevertheless sufficient to be toxic to R2J-GS cells. Moreover, selenite induced oxidative stress, as repeatedly shown by us and others, which may participate in R2J-GS cell death. Indeed, our study confirmed the mobilization of the GSH cellular pool in vitro via the decrease in the GSH/GSSG ratio. Interestingly, this ratio was unchanged in both the toxicological and tumoral regression studies, in which the pool of thiols was also unchanged in the plasma, brains, and livers of SS-treated mice.

The variation in the uptake and retention of Se regarding the tissues is consistent with the study of Burk et al. [44], and reflects the excretion pathways of Se. Indeed, excretory metabolites are eliminated through urine, feces, and the lungs to diminish accumulation outside the regulated Se pool, which is mainly controlled by the liver [45]. Nevertheless, the increase in Se in the brains of mice treated with SS, without notable adverse effects, is encouraging to the pursuit of our efforts. In the brains of camels supplemented with 8 mg/day for 70 days, an increase in Se was also observed [46]. 

The role of ROS as a key factor inducing apoptosis in cancer cells has been largely proven, supporting the notion that ROS are a critical driver of selenite-induced apoptosis in cancer cells [18,19]. Several signaling pathways have been explored, and among them, the AKT/β catenin pathway is triggered by ROS to induce apoptosis in colorectal cancer cells, similarly to the JNK/ATF2 pathway in NB4 cells, all of which belong to the MAPK family.

The tumor regression study showed that tumor progression was slowed by SS treatment, but without reaching the level of statistical significance (*p* = 0.08 SS vs. control group). It is worth noting that the number of mice per group was small (*n* = 4), and the tumor size was heterogeneous. Thus, even if this result is encouraging, it is not possible to draw any definitive conclusions from it about the efficacy of SS against tumor progression. Nevertheless, Tian et al. showed that in SCID mice xenografted with PC3 or HI-LAPC prostate cancer cells and then treated IP with 2 mg/kg of SS 3 times/week, tumor growth was significantly slowed in HI-LAPC but not PC3 cells, whereas SS combined with radiation therapy (RT) enhanced the inhibitory effect of RT [47]. In nude mice xenografted with prostate cancer cells, SS delivered via IP injection at 1.5 mg/kg daily for 5 weeks showed a reduction in tumor volume vs. the control group, and this effect was largely enhanced when SS was associated with carmustine [48]. 

The tumor progression in the TMZ group was the same as that of the control group, which was not surprising, as R2J-GS cells expressed MGMT. Interestingly, the decrease in plasmatic GPX activity observed in mice treated with TMZ at 5 mg/kg was also noticed in a patient GBM cell line treated with 100 µM TMZ [49]. These data could provide new information on the mechanism of action of TMZ. 

## 4. Materials and Methods

### 4.1. Cell Culture

R2J cells were isolated from surgical samples of an adult GBM patient who had undergone partial surgical resection at the Grenoble Hospital (France), after receipt of informed consent, as previously described [8].

The R2J cell line was firstly cultured in RPMI 1640 medium containing 10% fetal calf serum (FCS) supplemented with penicillin (100 IU/mL), streptomycin (100 µg/mL, PS), and L-Glutamine (2 mM) (Life Technologies), in a humidified hypoxia incubator (3% O_2_, 5% CO_2_, 37 °C). R2J cells were harvested using 0.5%-Trypsin-EDTA (10×) (#15400-054, Life Technologies).

R2J gliospheres (GS) were obtained by cultivating R2J cells in DMEM-F12 medium (Life Technologies), supplemented with epidermal growth factor (EGF, #130-093-825) and basic fibroblast growth factor (FGFβ, #130-093-564), both at 20 ng/mL, and 1X NeuroBrew-21 (#130-093-566)—all from Miltenyi Biotec—in a low-attachment-surface 10 cm Petri dish (Sarstedt, #83 3902500). R2J-GS cells were also grown in the hypoxic incubator.

As reported previously, R2J-GS cells express the stem cell markers CD44 and nestin, as in the parental tumor. Their tumorigenic potential has been assayed and validated by intracranial xenograft into athymic NudeFoxn1nu mice [8].

Sodium selenite (Sigma, #S5261) was dissolved in sterile water to obtain a 1 mM preparation. This solution was kept at 4 °C for 1 month.

TMZ (Temodal^®^) was dissolved in DMSO (10 mg/mL, i.e., 51.5 mM solution) and kept in the dark at RT for one month.

### 4.2. Selenium Uptake

R2J-GS cells were treated with SS for 24 h. Media were kept, and cells were subjected to five thaw/freeze cycles in Tris buffer (0.02 M, pH 7.4). Se content was determined both in cell lysates and in media, using ICP–MS (X series II, Thermo Fisher Scientific) as previously described [50]. The 78Se and 80Se isotopes were monitored. The recovery of Se in cell lysates and media was compared to the quantity of Se added. Se measured in nanomoles in cell lysates was normalized by total protein (g/L) and expressed in nanomoles/g of protein.

### 4.3. Dosage of Proteins

The concentrations of proteins in cell lysates or in the media were determined using the BCA assay (Interchim, Montluçon, France). The absorption at 562 nm was measured with the Varioskan Flash (Thermo Fisher Scientific), using SkanIt software for the quantification.

### 4.4. Se(0) Measurement

Two milliliters of a 1 M sodium sulfite solution prepared in 10 mM ammonium citrate buffer (pH 7) was added to the samples in a closed vessel. The mixture was warmed at 90 °C in a water bath for 1 h under vigorous shaking. The solution was then left to cool and centrifuged (14,000 rpm, 10 min). The supernatant was collected, filtered through a 0.45 µm filter, and analyzed via anion-exchange HPLC–ICP–MS. The HPLC column used was a PRP-X100 (250 mm × 4.1 mm, 5 µm) (Hamilton, Bonaduz, Switzerland). The chromatographic separations were carried out using a Model 1200 HPLC pump (Agilent, Wilmington, DE, USA), and the ICP–MS was an Agilent 7500 cx (Tokyo, Japan). The mobile phase was 10 mM ammonium citrate buffer (pH 7). Elution was performed via an isocratic program for 20 min at a flow rate of 1 mL/min. The collision/reaction cell of the ICP–MS was pressurized with H_2_. The 76Se, 77Se, 78Se, 80Se, and 82Se isotopes were monitored. The preparation of the selenosulfate stock solution (1000 mg/L) used for calibration is described in detail elsewhere [51].

Analytical-reagent-grade chemicals were purchased from Sigma-Aldrich (Saint-Quentin Fallavier, France). Water (18 MΩ·cm) obtained with a Milli-Q system (Millipore, Bedford, MA, USA) was used throughout.

### 4.5. Real-Time PCR

To study the molecular plasticity of the R2J cells, we alternatively cultured them in adherent culture conditions (described above), and then R2J-GS cells were harvested with trypsin and seeded in non-adherent and serum-free conditions in gliospheres (seeding at 1.106 per 10 cm-plate) for 14 days, and again in adherent culture conditions for 7 and 14 days. At these endpoints, R2J-GS and R2J cells were harvested to perform RT-q-PCR experiments.

Total RNA extraction was performed with NucleoSpin RNA following the manufacturer’s recommendations, with RNase-free DNase treatment (#740955, Macherey Nagel). cDNA was reverse-transcribed from 1 µg of total RNA with the SuperScript III First-Strand Synthesis System, followed by RNase H (1 µL) treatment (#11752, Life Technologies). Real-time PCR was conducted using the QuantiTect SYBR Green RT-PCR kit (#204143, Qiagen) and the Stratagene 3005MxPro (Santa Clara, CA, USA). The primers (sequences in Supplementary Table S1, Life Technologies) were all used at 400 nM, Tm at 60 °C, with the following qPCR program: 1 cycle: 15 min, 95 °C; 40 cycles: 15 s, 94 °C; 30 s, 60 °C; 30 s, 72 °C; 1 cycle: 1 min, 95 °C; 30 s, 60 °C; 30 s, 95 °C.

Gene expression was quantified using the comparative threshold cycle (Ct) method [52], with normalization against HPRT1, RPL27, and RPL32.

### 4.6. Quantitative Determination of Oxidized (GSSG) and Reduced (GSH) Glutathione Levels

After SS or TMZ treatments, R2J-GS cells were harvested. The pellets were obtained after centrifugation (3 min, 320 g, RT) and resuspended in isotonic Tris–HCl buffer (20 mM, pH 7.4, 300 mOsm), centrifuged (3 min, 320 g, RT), and rinsed twice with the Tris–HCl buffer. Then, cells were lysed in hypotonic Tris–HCl buffer and subjected to five freeze/thaw cycles. For glutathione determination, aliquots were taken from the whole homogenized lysate, deproteinized by adding an aqueous solution of 6% metaphosphoric acid, and centrifuged (2665 g, 4 °C, 10 min). Total glutathione (GSHt) was determined as described by Akerboom and Sies [53], and based on the spectrophotometric evaluation of the reduction rate of 5,5′-dithiobis-2-nitrobenzoicacid (DTNB, Sigma) into 5-thio-2-nitrobenzoic acid (TNB). Values were determined by comparing the reduction rate against a standard curve of glutathione. GSSG was measured under the same conditions after adjusting pH with ethanolamine and trapping the reduced glutathione (GSH) by adding 3-vinyl pyridine to the sample. GSSG and GSH were expressed as micromoles per gram of total cell protein, and the GSSG/GSH ratio was normalized to the controls (untreated R2J-GS cells).

Protein concentration in the lysates was determined using the BCA (bicinchoninic acid) protein assay reagent (#23225, Thermo Fisher), as described above.

### 4.7. Immunohistochemistry

A cytoblock was performed with dissociated R2J-GS cells fixed in 4% formol for 30 min and prepared with Shandon™ Cytoblock™ Reagent (Fisher scientific). The brains of mice were also fixed in 4% formol, and the cytoblocks were embedded in paraffin. HES and IHC analyses were performed from 3 µm paraffin sections using the Histostain^®^ Plus Kit and the Bond Polymer Refine Detection Kit (Leica DS9800) (Leica Biosystems, Newcastle, UK).

Anti-Olig2 (HPA003254) is a Sigma Life Science product. Anti-GFAP (ab33922), anti-CD44 (ab51037), anti-CD34 (ab110643) anti-nestin (ab93666), and anti-NCAM1 (ab75813) were obtained from Abcam (Cambridge, UK). Slides were incubated for 1 h with diluted antibodies (1/2000 for Olig2, 1/1000 for GFAP, 1/100 for CD44, 1/6400 for CD34, 1/200 for Nestin, 1/2000 for CD56) at room temperature before peroxidase revelation according to the Leica protocol. Pictures were captured using a Leica ICC50 camera connected to a Leica DM250 microscope (objective x20).

### 4.8. Cellular Invasion Assay

Invasion of R2J-GS cells was determined using a collagen-I-coated transwell insert (8 µm pore polycarbonate membrane (Corning, #003422A)) placed in a 24-well tissue culture plate. Coating was performed with 200 µL of collagen I, for 2 h, in the incubator. Then, collagen I was removed and 1.105 R2J-GS cells were prepared in 200 µL EGF/FGF-free medium and deposited on the insert, without (control) or with SS (5, 10, or 20 µM), for 48 h. The bottom well contained 600 µL of EGF/FGF-enriched medium (as described above) to incite cells to migrate.

Cells in the bottom well were fixed with 800 µL of 70% ethanol and stained with 0.2% crystal violet. Cells were then counted in five different quadrants with a Malassez cell. Results were expressed as the percentage of cells vs. the control (untreated cells).

### 4.9. Flow Cytometry

R2J-GS cells were seeded at 1 × 10^6^ cells in 10 cm low-adherence Petri dishes 24 h before treatment with SS for 24 h, after which cells and medium were recovered and centrifuged (3 min, 360 g, RT), and cells were rinsed twice with PBS1X.

Apoptosis and the cell cycle were evaluated with the FITC-Annexin V Apoptosis Detection Kit and the Cycle Test Plus DNA Reagent Kit (BD Biosciences, #556547), in accordance with the manufacturer’s instructions. The analysis of cells in the subG1 phase was used to determine DNA fragmentation.

Cell fluorescence was detected with a FACSCanto II (BD) and analyzed using FACS Diva software.

### 4.10. Soft Agar Cell Culture to Test Secondary Sphere Formation after SS and TMZ Treatments

R2J-GS cells were treated with SS (2.5, 5, 10, 100, and 200 µM) or TMZ (500 µM) for 24 h.

They were then harvested, gently dissociated by mechanical flushing, and counted under a microscope on a Malassez cell.

Agar (Sigma, St. Louis, MO, USA) was dissolved in complete medium to 1% to form the bottom layer; 1.8 mL of this mixture was deposited in each well of a 6-well plate to form a semi-solid feeding layer. A total of 1000 cells were then mixed with the 0.7% agar, dissolved in DMEM, and layered on top of the feeding layer. The cells were allowed to grow in the humidified and hypoxic incubator for 2 weeks before cell proliferation was evaluated via crystal violet coloration (0.4% in ethanol, 300 µL/well) and counting under the microscope. Results were reported as the number of colonies formed in the control vs. the experimental conditions, and expressed as the percentage of the control.

### 4.11. In Vivo Experiments

All animal protocols were approved by the GIN Animal Care and Use Committee (number 2017121517085709).

### 4.12. Delimitation of SS Toxic Doses

**Figure 5:**
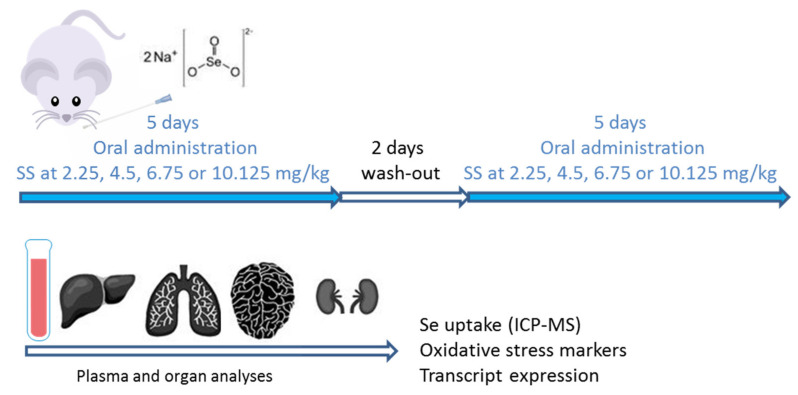
Experimental design to study the toxicity of SS in BALB/c mice.

Before studying tumor regression caused by SS treatment, we launched a toxic dose study (Figure 5) to delineate the SS dose acceptable by the mice (BALB/cOlaHsd, male, 8 weeks, Envigo, France), according to the experimental design: 5 days on, 2 days off, and 5 days on. On days on, SS was given orally by gavage using plastic feeding tubes (Phymep, #FTP-20-30). An escalation dose study was performed at 2.25, 4.5, 6.75, and 10.5 mg/kg (*n* = 3 mice per dose). SS solution was prepared as mentioned for cell culture experiments. TMZ (20 mg, Mylan) was dissolved in a monohydrate citric acid solution (7 mg/mL) to obtain a solution at 1 mg/mL, as described in [54]. Aliquots (500 µL) were prepared and stocked at −20 °C. One aliquot per day was used to treat the mice.

After the treatments, mice were deeply anesthetized (10 mg/kg xylazine and 75 mg/kg ketamine), 300 µL of blood was harvested by cardiac puncture, and mice were perfused with PBS. Blood was immediately centrifuged in heparin tubes (4000× *g*, 10 min, RT). Brains, lungs, kidneys, and livers were removed. Some of the organs were immediately fixed in 4% formol for histopathological analyses, while the rest were snap-frozen for biochemical and molecular analyses.

### 4.13. Tissue Preparations

For Se analysis, organs were weighed and immediately mineralized at atmospheric pressure in 67% HNO3 for 24 h at room temperature, and then for 48 h in the oven at 60 °C. Then, the samples were treated as described above.

For transcript expression, organs were weighed and homogenized in NucleoZOL (#740404, Macherey-Nagel), followed by extraction with NucleoSpin RNA for NucleoZOL (#740406, Macherey-Nagel), as recommended. Then, the samples were treated as described above.

For antioxidant analyses, organs were weighed, and then homogenized in cold Tris 5 mM-DTPA 0.5 mM buffer (pH 7.4) enriched with 0.2% PMSF (0.5 M); 100 mL of this buffer was necessary for 100 mg of tissue. The homogenates were centrifuged at 3000× *g* for 10 min at 4 °C.

The resulting supernatant was used for the analyses of GPX1 activity; GSH, GSSG, MDA, and thiol groups; and to determinate the protein concentration as described above.

For IHC, organs were fixed in 4% formol and then embedded in paraffin, sliced, and stained with hematoxylin and eosin, before proceeding to specific protein immunodetection as described above.

### 4.14. Determination of Malondialdehyde (MDA)

The evaluation of MDA in plasma is based on its reaction with thiobarbituric acid (TBA) using reversed-phase high-performance liquid chromatography (HPLC). The MDA–TBA adducts were separated from interfering substances, and the breakdown product 1,1,3,3-tetraethoxypropane (TEP) was employed as a standard. TEP undergoes hydrolysis to liberate stoichiometric amounts of MDA. Stock standard solution (480 mL of TEP in 100 mL ethanol) was prepared, and this primary solution was diluted to concentrations of 0, 1, 2, 3, 4, 5, and 6 mM. Tissue extract aliquots or standards were mixed with TBA (0.8%) and placed in a water bath at 95 C for 1 h, and then cooled. Samples were neutralized with a methanol–NaOH mixture (pH 6.0), and protein-free supernatants were chromatographed in the HPLC system after centrifugation. The column used for the separation was an Adsorbosphere C18 (5 mm particle diameter, 250 mm, 4.6 mm ID). The MDA–TBA adducts were eluted with potassium dihydrogen phosphate buffer (10 mM, pH 6.0)–acetonitrile (17%). The absorption of MDA equivalents generated by the acid-catalyzed hydrolysis of TEP generated a standard curve that allowed for the quantification of MDA derivatives.

### 4.15. GPX1 Determination

GPX1 activity was measured using the GR-NADPH method. Activity was determined via a coupled-assay system.

Glutathione peroxidase-1 (GPX1) activity was determined via the method of Flohe and Gunzler [55], with some modifications. The rate of glutathione oxidization by tert-butyl hydroperoxide (30 mM) was evaluated by the decrease in NADPH2 (8.4 mM in Tris buffer) at 340 nm in the presence of EDTA (1 mM), excess reduced glutathione (0.15 M in Tris buffer), and glutathione reductase (200 U/mL in Tris buffer). GPX1 activity was expressed as international units per gram of soluble cell proteins

### 4.16. Thiol Group Evaluation

Protein oxidation was evaluated by determination of thiol groups (SH) via a colorimetric method based on the reducing properties of the SH groups [56]. The disulfide bridge (SS) obtained in Ellman’s reagent or DTNB is reduced in presence of SH groups, and produces aromatic sulfhydryl derivatives (TNB) colored in yellow, whose absorbance was measured at 412–415 nm. SH levels were calculated by projection on a standard curve and expressed as µmol/g of total cell protein, and then expressed as the percentage vs. the control.

### 4.17. Striatum R2J-GS Xenograft into Nude Mice and Treatment with SS or TMZ

**Figure 6:**
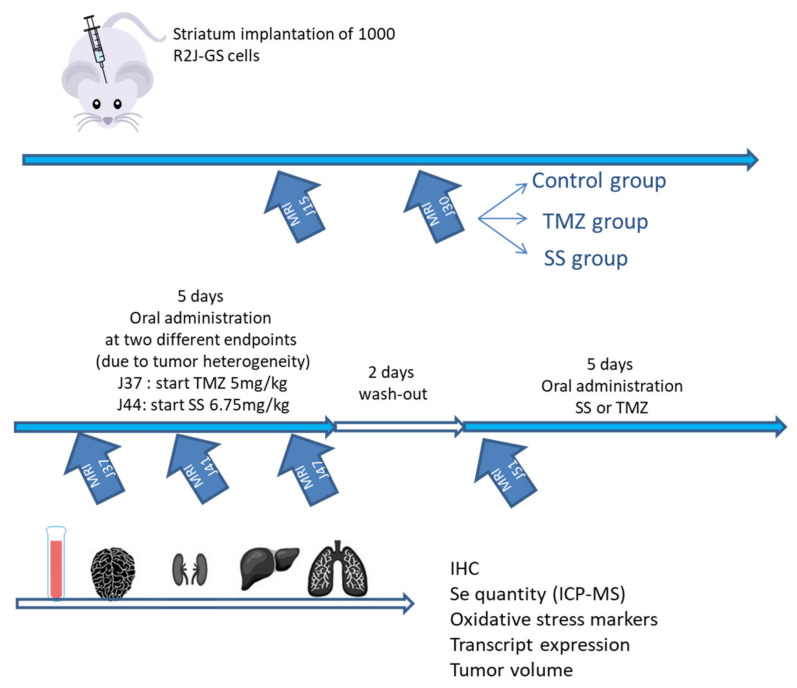
Experimental design to study the tumor regression in nude mice implanted with R2J-GS cells.

R2J-GS cells (n = 1000) were dissociated before being implanted into the striata of 4–6-week-old athymic NudeFoxn1nu male mice (n = 16, Envigo), as previously described [8]. We showed that such an implantation takes ~32 days to develop visible and quantifiable tumors in nude mice [8]. The experimental design is presented in the Figure 6.

After the second magnetic resonance imaging (MRI, J30 PI), the mice were separated into 3 groups, according to tumor size and the presence or absence of ventriculitis, to constitute comparable experimental groups (control, SS, and TMZ (n = 4 mice/group)).

The design of the treatment with TMZ or SS was as follows: 5 days with oral gavage, 2 days off, and 5 days with oral gavage using plastic feeding tubes, as described in the toxicological study.

We decided, due to tumor heterogeneity, to start the treatments of TMZ (5 mg/kg) and SS (6.75 mg/kg) at two different timepoints. For TMZ, the treatment started at D37 to D41, followed by 2 days off, and then resumed from D44 to D48 PI. For SS, the treatment started at D44 to D48, followed by 2 days off, and then resumed from D51 to D55 PI.

After the treatments, mice were perfused, and their brains, lungs, kidneys, and livers were removed. Some of the organs—and the brains, transversally cut at the level of the tumor—were immediately fixed in 4% formol for histopathological analyses, while the rest were snap-frozen for molecular analyses.

### 4.18. Magnetic Resonance Imaging (MRI)

MRI sessions were performed 15, 30, 37, 41, 47, and 51 days PI.

MRI sessions were achieved at 7T (Avance III console; Bruker, Ettlingen, Germany; IRMaGe MRI facility, Grenoble, France) using an actively decoupled cross-coil setup (volume coil for radiofrequency transmission and surface coil for signal reception). All MRI experiments were performed under anesthesia: 5% isoflurane for induction, and 2% for maintenance in air. Mice were maintained at 37.0 °C and their breath rate was maintained at 60 breaths/min throughout the acquisition. After shimming, MRI sequences were designed for the acquisition of anatomical T2-weighted (T2W) images using a spin-echo MRI sequence (TR/TE = 2900/42 ms, NA = 14, 21 slices with field of view (FOV) 20 × 20 mm^2^, matrix = 256 × 256 and voxel size = 156 × 156 × 500 μm^3^). The acquisition duration was 10 min and 50 s. Tumor volume was calculated using a 3D slicer (V4.10.2).

### 4.19. Statistical Analysis

Data are presented as means and standard deviations (SD) of at least three independent experiments. Statistical significance was determined using Student’s *t*-test or ANOVA when appropriate, using StatView and GraphPad Prism (V5) software to generate the illustrations with * *p* < 0.05; ** *p* < 0.01; *** *p* < 0.005, and $ *p* < 0.0001.

## Data Availability

Not applicable.

## Supplementary Materials

The following are available online at https://www.mdpi.com/article/10.3390/ijms221910646/s1.
