# Supplementary material for: Preclinical Evaluation of Sodium Selenite in Mice: Toxicological and Tumor Regression Studies after Striatum Implantation of Human Glioblastoma Stem Cells
Source: Int J Mol Sci. 2021 Sep 30;22(19):10646. doi: 10.3390/ijms221910646 (PMC8508933; doi:10.3390/ijms221910646)
Supplement: Supplementary file 1 [file ijms-22-10646-s001.zip › ijms-1371328-supplementary.pdf]

Table S1. sequences of the primers used in this study and size of the PCR product obtained.

Name	Forward (5′-3′)	Reverse (5′-3)′	Product size (pb)
CD34	CTACAACACCTAGTACCCTTGGA	GGTGAACACTGTGCTGATTAC A	185
CD44	CTGCCGCTTTGCAGGTGTA -	CATTGTGGGCAAGGTGCTATT	109
GALC	TATTTCCGAGGATACGAGTGGT	CCAGTCGAAACCTTTTCCCAG	117
GPX1	AGTCCACCGTGTATGCCTTCT	GAGACGCGACATTCTCAATGA	105
MMP2	CCGTCGCCCATCATCAAGTT	AAACAGGTTGCAGCTTCTCCT	114
Nestin	CTGCTACCCTTGAGACACCTG	GGGCTCTGATCTCTGCATCTAC	141
SEPP1	AGCTCTGCTTGTTACAAAGCC	CAGGTCTTCCAATCTGGATGC	144
SOX2	TGGACAGTTACGCGCACAT	CGAGTAGGACATGCTGTAGGT	215
TUBB3	GGCCTCTTCTCACAAGTACG	GAAGAGATGTCCAAAGGCCC	97
RPL27	TGATGGCACCTCAGATCGC	AGAGTACCTTGTGGGCATTAG G	240
RPL32	TTAAGCGTAACTGGCGGAAAC	GAGCGATCTCGGCACAGTAA	210
